# Supplementary material for: Identification of Freezing-Responsive microRNAs and Their Targets in Chinese Jujube by Small RNA and Degradome Sequencing
Source: Int J Mol Sci. 2026 Sep 4;27(17):7912. doi: 10.3390/ijms27177912 (PMC13566890; doi:10.3390/ijms27177912)
Supplement: Supplementary file 1 [file ijms-27-07912-s001.zip › Supplementary Material S2/novel_miR_27.pdf]

Provisional ID	:	novel_miR_27
Score total	:	9286.7
Score for star read(s)	:	3.9
Score for read counts	:	9280.5
Score for mfe	:	0.7
Score for randfold	:	1.6
Score for cons. seed	:	
Total read count	:	18215
Mature read count	:	18165
Loop read count	:	0
Star read count	:	50

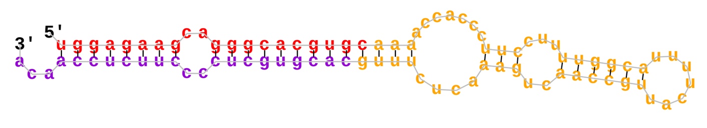

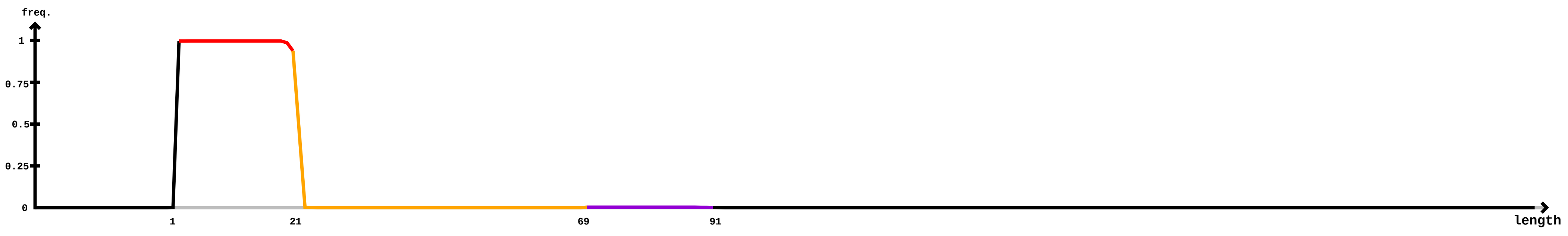

Mature **Star**

```
5'- uuuuuggugugguagcuccuuguuggagaagcagggcacgugcaaaaccacccuuccuuuuggcauuuucauugccaacugaaacucuuugcacgugcuccccuucuccaacauggguuccucaccuuugucccucuauguuuaaccaccauuguauuauauauaaucuuagguaaugcaguaucaguacucuuuguuuuucuauauccucucugcuuucaguuucugauuaacauuauuuauuuauuuac -3' obs
    uuuuuggugugguagcuccuuguuggagaagcagggcacgugcaaaaccacccuuccuuuuggcauuuucauugccaacugaaacucuuugcacgugcuccccuucuccaacauggguuccucaccuuugucccucuauguuuaaccaccauuguauuauauauaaucuuagguaaugcaguaucaguacucuuuguuuuucuauauccucucugcuuucaguuucugauuaacauuauuuauuuauuuac     exp
    .....((((.(.(((.((.(((((((((((..((((((((((((.......(((...((((((.......))))))..)))....))))))))))))..))))))))))).))))).).))))..........(((((........((((((((((.((......)).))))))))))(((((.(((....((................))....)))..))))).)))))...............
```

read	reads	mm	sample
Cuggagaagcagggcacgu	1	1	LT-1
Cuggagaagcagggcacgugc	1	1	LT-2
uuggagaagcagggcacgugc	3	0	JCK-2
Cuggagaagcagggcacgugc	4	1	JCK-3
uuggagaagcagggcacgugc	3	0	LT-3
uuggagaagcagggcacgugc	1	0	LT-1
Cuggagaagcagggcacgugc	3	1	JCK-2
uuggagaagcagggcacgugc	1	0	JCK-3
Cuggagaagcagggcacgugca	1	1	LT-1
Cuggagaagcagggcacgugca	3	1	JCK-3
Cuggagaagcagggcacgugca	1	1	LT-3
uggagaagcagggcacgu	21	0	LT-2
uggagaagcagggcacgu	19	0	LCK-3
uggagaagcagggcacgu	16	0	JCK-2
uggagaagcagggcacgu	34	0	LT-3
uggagaagcagggcacgC	1	1	LT-3
uggagaagcCgggcacgu	1	1	JCK-2
uggagaagcagggcacgu	33	0	LT-1
uggagaagcagggcacgu	9	0	JCK-3
uggagaagcagggcacgu	31	0	JT-2
uggagaagcagggcacgu	2	0	JCK-1
uggagaagcagggcacgu	26	0	JT-3
uggagaagcagggcacgug	76	0	JT-3
ugAagaagcagggcacgug	1	1	LT-2
uggagCagcagggcacgug	1	1	LT-1
uggagaagcagggNacgug	1	1	LCK-1
uggagaagcagggcacgug	66	0	LT-1
uggagaagUagggcacgug	1	1	LT-1
uggagaagcagggcacgug	41	0	JCK-3
uggagaagcagggcacgug	127	0	LCK-1
uggagaagcagggcacgug	49	0	LCK-3
uggCgaagcagggcacgug	1	1	LT-2
uggagaagcagggcacguA	2	1	JT-3

Mature Star

uuuuggugguguagcuccuuguuggagaagcagggcacgugcaaaaccacccuuccuuuuggcauuuucauugccaacugaaacucuuugcacgugcuccccuucuccaacauggguuccucaccuuugucccucuauguuuaaccaccauuguauuauauauaaucuuagguaaugcaguaucaguacucuuuguuuuucuauauccucucugcuuucaguuucugauuaacauuauuuauuuauuuac			
.....................uggagaagcagggcacgug...	122	0	LCK-2
.....................uggagaagcagggcacgug...	109	0	LT-2
.....................uggagaagcCgggcacgug...	1	1	LT-2
.....................uggagaagcagggcacguC...	3	1	LT-3
.....................uggagaagUagggcacgug...	1	1	JCK-2
.....................uggagaagcagggcacgug...	122	0	LT-3
.....................uggagaagcagggcacgug...	53	0	JCK-2
.....................uggagaagcagggcacguA...	1	1	LT-3
.....................uggagaagcagggcacgug...	54	0	JT-2
.....................uggagaagcaUggcacgug...	1	1	JT-3
.....................uggagCagcagggcacgug...	1	1	JT-3
.....................uggagaagcagggcacgug...	37	0	JCK-1
.....................uggCgaagcagggcacgug...	1	1	LT-3
.....................uggNgaagcagggcacgug...	1	1	JCK-1
.....................uggagaagcagggcacguA...	7	1	LT-2
.....................uggagaagcagggcaAgug...	1	1	LT-2
.....................uggagaagcCgggcacgugc..	3	1	LT-2
.....................uggagaagcagggcCcgugc..	3	1	JCK-1
.....................uggagaagcagggcCcgugc..	1	1	LT-1
.....................uggagaagcagggcacgugc..	1129	0	JCK-3
.....................uggagaagcCgggcacgugc..	1	1	JCK-2
.....................uggagaagUagggcacgugc..	2	1	JT-3
.....................uggCgaagcagggcacgugc..	1	1	JT-2
.....................uggagaagcCgggcacgugc..	1	1	JCK-1
.....................uggagaagcagggcCcgugc..	1	1	JT-2
.....................uggagaCgcagggcacgugc..	7	1	LT-3
.....................uggagGagcagggcacgugc..	1	1	LCK-1
.....................uAgagaagcagggcacgugc..	1	1	JCK-3
.....................uggCgaagcagggcacgugc..	1	1	LT-1
.....................uggagaagcagggcacgugU..	2	1	JT-3
.....................uNgagaagcagggcacgugc..	1	1	LT-1
.....................uggagaagcagggcacgugc..	1625	0	JCK-2
.....................uggagGagcagggcacgugc..	1	1	LT-1
.....................uNgagaagcagggcacgugc..	1	1	JCK-3
.....................ugNagaagcagggcacgugc..	1	1	LCK-1
.....................uggagCagcagggcacgugc..	3	1	LT-1
.....................uggaAaagcagggcacgugc..	1	1	LT-3
.....................ugAagaagcagggcacgugc..	1	1	LCK-3
.....................uggagaagcagggcCcgugc..	2	1	LT-2
.....................uggNgaagcagggcacgugc..	1	1	JCK-1
.....................uggagaagcagggcacguAc..	1	1	JCK-2
.....................uggagaagUagggcacgugc..	1	1	LT-1
.....................uggaAaagcagggcacgugc..	1	1	JT-3
.....................uggagaagcagggcacgugU..	44	1	LT-2
.....................uggagaagUagggcacgugc..	10	1	JCK-2
.....................uggagaagcCgggcacgugc..	1	1	JCK-3
.....................uAgagaagcagggcacgugc..	1	1	JCK-1
.....................uggagaagcagggcGcgugc..	1	1	LCK-1
.....................uggagaagcagggcacgugU..	4	1	JCK-1
.....................uggCgaagcagggcacgugc..	1	1	JCK-3
.....................uggagaagcagNgcacgugc..	2	1	JCK-1
.....................uggagaagcagggcaUgugc..	2	1	LT-3
.....................uggagCagcagggcacgugc..	1	1	JCK-3
.....................uggagaCgcagggcacgugc..	1	1	JT-2
.....................Gggagaagcagggcacgugc..	1	1	LCK-2
.....................uggagaagAagggcacgugc..	1	1	JCK-2
.....................uggagaagcCgggcacgugc..	5	1	LT-3
.....................uggagaagcGgggcacgugc..	2	1	LT-3
.....................uggagaagcagggcaUgugc..	2	1	JCK-2
.....................uggagaagcagggcacgugc..	1369	0	LT-2
.....................ugAagaagcagggcacgugc..	1	1	JCK-3
.....................uggagaCgcagggcacgugc..	1	1	LCK-2
.....................uggagaagcagggcacgugN..	1	1	JCK-1
.....................uggagCagcagggcacgugc..	4	1	LT-3
.....................uggagCagcagggcacgugc..	1	1	LT-2
.....................uggagaagcagggcCcgugc..	1	1	JCK-3
.....................ugAagaagcagggcacgugc..	2	1	LT-2
.....................uggagaagcagggcacgugc..	604	0	JT-2
.....................uggagCagcagggcacgugc..	2	1	JCK-2
.....................uggagaagcagggcCcgugc..	1	1	LCK-2

Mature **Star**

uuuuugguguguagcuccuugu**uggagaagcagggcacgugc**aaaaccacccuuccuuuuggcauuuucauugccaacugaaacucuuug**cacgugcuccccuucuccaaca**uggguuccucaccuuugucccucuauguuuaaccaccauuguauuauauauaaucuuagguaaugcaguaucaguacucuuuguuuuucuauauccucucugcuuucaguuucugauuaacauuauuuauuuauuuac

uggagaagcagggcacgugc	875	0	JCK-1
uggagaagcagggcCcgugc	1	1	JT-3
uggagaagUagggcacgugc	3	1	LT-2
uggagaagcagggcCcgugc	3	1	JCK-2
uggagaagUagggcacgugc	2	1	JCK-1
uggCgaagcagggcacgugc	11	1	LT-2
uggagaCgcagggcacgugc	2	1	LT-1
uggagaagcagggUacgugc	1	1	JCK-3
uggagaagcagggcacgugU	11	1	LCK-3
uAgagaagcagggcacgugc	1	1	LT-1
uggagaagcagggcacgugU	25	1	LT-1
uggagaagcagggcNcgugc	2	1	LCK-1
uggagaagcagAgcacgugc	1	1	LT-3
uggagaagcagggcacgugU	11	1	JCK-3
uggagaCgcagggcacgugc	1	1	JT-1
uggCgaagcagggcacgugc	4	1	LT-3
uggagaagUagggcacgugc	7	1	JCK-3
uggagaagcagggcacgugc	319	0	LCK-1
uggagaagcagggcaUgugc	1	1	JCK-1
uggCgaagcagggcacgugc	3	1	JCK-2
uggagaagcagggcacgugc	2380	0	LT-3
uggagaagcagggcCcgugc	3	1	LT-3
uggagaagcagggcacgugc	318	0	LCK-2
uggagCagcagggcacgugc	2	1	JCK-1
uggagaagcagggcacgCgc	1	1	LT-3
ugNagaagcagggcacgugc	3	1	LT-2
uggagaagcagggcacgugU	16	1	LT-3
uggagaagcagggcacgugc	857	0	JT-3
uggagaCgcagggcacgugc	3	1	JCK-3
uggagaagUagggcacgugc	3	1	LT-3
uggagaagcagggNacgugc	1	1	LCK-1
uggagaagcagggNacgugc	1	1	JT-2
uggagaagcagggcacgugc	532	0	LCK-3
uggagaagcCgggcacgugc	1	1	JT-2
uggCgaagcagggcacgugc	1	1	LCK-1
Cggagaagcagggcacgugc	2	1	LT-2
uggagaagcagggcacgugU	21	1	JCK-2
uggNgaagcagggcacgugc	2	1	JT-2
uggagaagcCgggcacgugc	1	1	LCK-3
uNgagaagcagggcacgugc	1	1	LCK-1
uggagaCgcagggcacgugc	6	1	JCK-2
uUgagaagcagggcacgugc	1	1	JCK-3
uNgagaagcagggcacgugc	2	1	JCK-2
uggagaagcagggcacgugc	2130	0	LT-1
uggagaagcCgggcacgugc	1	1	LT-1
uAgagaagcagggcacgugc	4	1	LT-2
uggGgaagcagggcacgugc	1	1	LCK-3
Cggagaagcagggcacgugc	1	1	JCK-3
uggagaagcaggAcacgugc	1	1	LCK-3
Cggagaagcagggcacgugc	1	1	LT-3
uggCgaagcagggcacgugc	1	1	JCK-1
uggagaagcagggcacgugc	121	0	JT-1
uggagaCgcagggcacgugc	3	1	LT-2
uggagaCgcagggcacgugca	1	1	LT-3
uggagaagcagggcGcgugca	1	1	JCK-3
uggagaagcagggcacgugca	360	0	LT-1
uggagCagcagggcacgugca	1	1	JCK-2
uggagaagcagggcacgugca	2	0	LCK-2
uggagaagcagggcacgugUa	1	1	LT-1
uggCgaagcagggcacgugca	1	1	JCK-1
uggagaagUagggcacgugca	1	1	JT-1
uggagaagcCgggcacgugca	1	1	JCK-1
uggagaagcagggcacgugcU	10	1	LT-2
uggagaCgcagggcacgugca	1	1	JCK-3
uggagaagcagggcacgugcU	16	1	JT-1
uggagaagcagggcacgugca	100	0	JT-1
uggagaagcagggcacgugNa	1	1	JCK-1
uggagaCgcagggcacgugca	3	1	JCK-2
uggagaagcagggcacgugcG	1	1	JCK-2
uggagaagcagggcCcgugca	1	1	JCK-3

Mature Star

uuuuggugugguagcuccuuguuggagaagcagggcacgugcaaaaccacccuuccuuuuggcauuuucauugccaacugaaacucuuugcacgugcuccccuucuccaacauggguuccucaccuuugucccucuauguuuaaccaccauuguauuauauauaaucuuagguaaugcaguaucaguacucuuuguuuuucuauaucccucucugcuuucaguuucugauuaacauuauuuauuuauuuac			
uNgagaagcagggcacgugca	1	1	JCK-3
uggagaagcagggcacgugca	143	0	LT-2
uggagaagcagggcaUgugca	1	1	JCK-2
uggagaagcagggcacgugca	58	0	LCK-3
uggagaCgcagggcacgugca	1	1	JCK-1
uggagaagcagNgcacgugca	2	1	JCK-1
uggagaagcagggcacgugcC	7	1	LT-1
uggagaagcagggcacgugcU	6	1	LT-3
uNgagaagcagggcacgugca	5	1	JCK-2
uggagaagcagggcacgugca	317	0	LT-3
uggagaagcagggcacgugcU	19	1	LCK-3
uggagaagcagggcaGgugca	1	1	JCK-2
uggagaagcagggcacgugca	805	0	JCK-1
uggCgaagcagggcacgugca	2	1	LT-1
uggagaagcagggcacgugUa	4	1	LT-2
uggagaagcagggUacgugca	1	1	LT-1
uggagaagcagggcaAgugca	1	1	JCK-1
uggagaagcagggcacgugcU	1	1	JCK-1
uggagaCgcagggcacgugca	1	1	JT-3
uggagaagcagggcacgugca	1285	0	JCK-2
uggagaagcagggcacAugca	1	1	JT-3
uggagaagcagggcacgugcC	2	1	JCK-1
uggagCagcagggcacgugca	1	1	LT-2
uggagaagcagggcCcgugca	1	1	JCK-2
uAgagaagcagggcacgugca	1	1	JCK-3
uggagaagcagggcacAugca	2	1	JT-2
uggagaagcagggcaGgugca	1	1	JCK-1
uggagaagcagggcacgugcC	4	1	JCK-3
uggagaagUagggcacgugca	1	1	LT-1
uggagaagUagggcacgugca	3	1	JCK-1
uggagaagcagggcacgugcU	13	1	LT-1
uggagaagUagggcacgugca	6	1	JCK-2
uggagaagcagggcCcgugca	1	1	LT-2
uggagaagcagggcCcgugca	1	1	LT-1
uggagCagcagggcacgugca	1	1	JCK-1
uggagaagcagggcacgugcC	3	1	LT-3
uggagCagcagggcacgugca	1	1	JCK-3
uggagaagcagggcacgugcC	4	1	LT-2
uggagaagcagggcacgugcC	9	1	JCK-2
uggCgaagcagggcacgugca	1	1	JCK-2
uggagaagcagggcacgugcU	7	1	JCK-2
uggagaagcagggcacgugca	92	0	JT-2
uggagaagcagggcacgugca	1008	0	JCK-3
uggNgaagcagggcacgugca	1	1	JCK-1
uggagaagcagggcacgugca	131	0	JT-3
uNgagaagcagggcacgugca	1	1	JCK-1
uggagaagUagggcacgugca	6	1	JCK-3
uggagaagcagggcacgugUa	1	1	LT-3
uggagaagcagggcacgugcaa	3	0	JCK-2
uggagaagcagggcacgugcaa	1	0	LT-1
uggagaagcagggcacgugcaG	1	1	LT-1
uggagaagcagggcacgugcaa	1	0	JCK-1
uggagaagcagggcacgugcaa	3	0	JCK-3
uggagaagcagggcacgugcaC	1	1	JCK-1
uggagaagcagggcacgugcaaa	21	0	JT-1
uggagaagcagggcacgugcaCa	2	1	LT-1
uggagaagcagggcacgugcaaa	1	0	LT-1
gagaagcagggcacgugc	2	0	JCK-3
cacgugcuccccuucucc	1	0	JCK-1
cacgugcuccccuucucca	6	0	LCK-3
cacgugcuccccuucucca	2	0	JCK-1
cacgugcuccccuucucca	3	0	JCK-2
cacgugcuccccuucuccaa	2	0	LT-3
cacgugcuccccuucuccaac	3	0	JCK-3
cacgugcuccccuucuccaaA	1	1	LT-1
cacgugcuccccuucuccaac	2	0	LT-1
cacgugcuccccuucuccaac	5	0	JCK-2
cacgugcuccccuucuccaac	2	0	JCK-1
cacgugcucUccuucuccaac	1	1	LT-3
cacgugcuccccuucuccaacU	2	1	JCK-3

```
                    Mature                                                              Star
uuuugguguguagcuccuuguuggagaagcagggcacgugcaaaaccacccuuccuuuuggcauuuucauugccaacugaaacucuuugcacgugcuccccuucuccaacauggguuccucaccuuugucccucuauguuuaaccaccauuguauuauauauaaucuuagguaaugcaguaucaguacucuuuguuuucuauauccucucugcuuucaguuucugauuaacauuauuuauuuauuuac
.........................................................................................cacgugcuccccuucuccaacU.........................................................................................................................................  3  1  LT-1
.........................................................................................cacgugcuccccuucuccaacU.........................................................................................................................................  3  1  LT-2
.........................................................................................cacgugcuccccuucuccaacU.........................................................................................................................................  8  1  JCK-2
.........................................................................................cacgugcuccccuucuccaacU.........................................................................................................................................  5  1  JCK-1
..........................................................................................acgugcuccccuucucca............................................................................................................................................  1  0  JCK-2
```
